# Impact of smart health systems on the behavior of older adults under community healthcare

**DOI:** 10.3389/fpubh.2022.1056817

**Published:** 2022-12-05

**Authors:** Jing Zhao, Liangyu Wang, Kaimeng Guo

**Affiliations:** ^1^Zhejiang Dongfang Polytechnic, Wenzhou, China; ^2^School of Management, North Sichuan Medical College, Nanchong, China; ^3^The Faculty of Social Sciences, University of Macau, Taipa, Macao SAR, China

**Keywords:** aging population, medical behavior, community healthcare, smart healthcare, public health

## Abstract

**Background:**

With the trend of world population aging, a good community health care system will determine whether the elderly can get good medical conditions. How to improve the community health care system can study how the behavior of the elderly affects it.

**Objective:**

This paper is based on the analysis of the current situation of population aging at home and abroad.

**Methods:**

On the premise of analyzing the demand and behavior of elderly people seeking medical treatment and the function of community health service institutions. Literature research was conducted to analyze the influencing factors of community health care needs and elderly people's medical seeking behavior at home and abroad. Then the elderly in Tianjin were investigated by issuing questionnaires, and the law of medical treatment behavior of the elderly in Tianjin was calculated. Combined with the results of relevant investigations abroad, the common phenomenon is summarized. Finally, the analysis method of intelligent medical system is proposed, and the design process of system acquisition module and user usage mode are given.

**Result:**

The smart medical system can bring great convenience to the elderly and community healthcare.

**Discussion:**

It emphasizes the powerful functions of smart health systems and their future importance for the health care of the elderly.

## Introduction

### Aging population

#### The aging trend of the world population

Population aging is an inevitable result of demographic transition and an important issue facing human society in the 21st century. Since the 21st century, the world as a whole has entered an aging society. The population aging degree of developed countries has been significantly deepened, and developing countries have not yet entered the ranks of aging. From a global perspective, in 2000, the total population of the world was 6.14 billion, which will increase to 7.79 billion in 2020, an increase of 1.65 billion in 20 years; at the same time, the population of people aged 60 and above has increased from 610 million to 1.05 billion people, an increase of 440 million people; in 2000, the proportion of the elderly population aged 60 and above was 9.9%, which is on the eve of an aging society. During the same period, the population of the elderly aged 60 and above in developed countries increased from 230 million to 330 million, an increase of 100 million, and the proportion of the total population increased from 19.5 to 25.7%, an increase of 6.2 percentage points. The degree of population aging is relatively deep and similar. Much higher than the world's overall level. The number of elderly people aged 60 and over in developing countries (excluding China) increased from 250 million to 470 million, an increase of only 220 million, and the proportion of the total population increased from 6.8 to 9.2%, an increase of 2.4 percentage points. The population is aging. The degree of aging is relatively mild and the process is significantly slower than that of the world, and it has not yet entered the aging society as a whole, as shown in Table 1.

**Table 1:** Population aging in the world and China from 2000 to 2020 (World Census Report).

**Classification**	**Total population/billion people**			**60**+ **population/billion people**			**60**+ **proportion of population/%**		
	**2000**	**2010**	**2020**	**2000**	**2010**	**2020**	**2000**	**2010**	**2020**
World	61.4	69.6	77.9	6.1	7.6	10.5	9.9	11.0	13.5
Developed countries	11.9	12.3	12.7	2.3	2.7	3.3	19.5	21.8	25.7
Developing countries (Excluding China)	36.4	43.2	50.5	2.5	3.2	4.7	6.8	7.5	9.2
China	12.6	13.4	14.2	1.3	1.8	2.6	10.0	13.3	18.7

The aging of the world's population shows the following trends (1).

➀ The world has entered the process of population aging, and developed countries are at the forefront. The pace of population aging in China is significantly faster than that of developed countries and faster than that of the world.
➁ Japan is the most aging country today, and China has not yet entered the ranks of countries with serious population aging. China's aging process is significantly faster than that of the United States, Germany, Russia and other countries, and slower than Japan, South Korea, and Finland.
➂ The population aging trend in developed countries is obvious. China's aging degree is heavier than the overall level of the world, but significantly lower than that of developed countries.
➃ The degree of aging in developed countries in Japan and Europe is generally relatively deep, while that in China is relatively light, and the trend of aging in developing countries such as India and Nigeria is not yet obvious.
➄ The aging process of the world's population continues to advance. Developed countries have entered a severely aging society, and developing countries will also enter an aging society. China's aging and low birthrate are developing rapidly.

#### The trend of population aging in China

**Figure 1:**
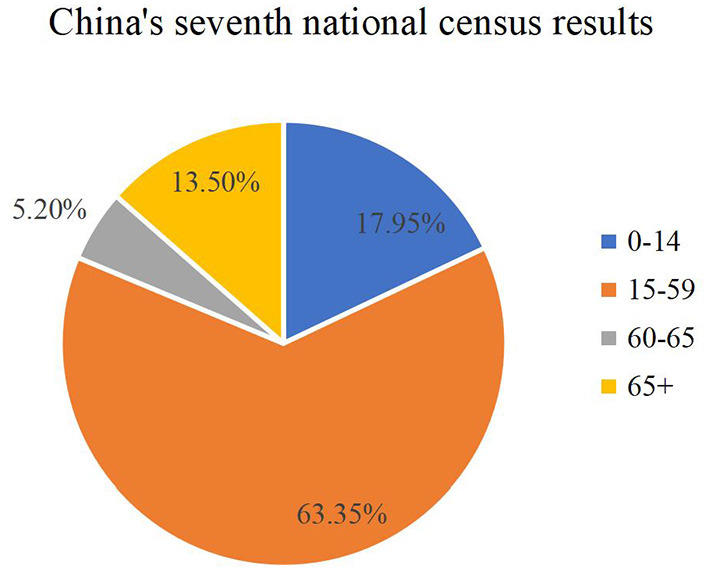
The results of the seventh national census (Sixth National Census Report).

The results of China's seventh national census show that the population aged 0–14 is 253.38 million, accounting for 17.95%; the population aged 15–59 is 894.38 million, accounting for 63.35%; the population aged 60 and above is 264.02 million, accounting for 18.70% % (including 190.64 million people aged 65 and over, accounting for 13.50%) as shown in Figure 1.

The population aging pressure during the “14th Five-Year Plan” period is greater than that during the “13th Five-Year Plan” period. The 1962–1976 baby boomer population will age in the next 5–10 years. It is expected to enter a super-aging society with a proportion of more than 20% around 2033, and then continue to rapidly rise to 35% in 2060.

China's population aging presents the following five characteristics (1).

➀ The size of the elderly population is huge.
➁ The speed of aging is fast.
➂ The problem of advanced age and empty nest is becoming more and more prominent.
➃ The elderly dependency ratio is rising sharply and the burden of old-age care is increasing.
➄ Aging before getting rich.

### Healthcare needs and behaviors of older adults

As we all know, with the increase of human age, the functions of all aspects of the body weaken, resulting in the elderly becoming a disease-prone population, especially chronic diseases, which have a long course of disease and require long-term follow-up treatment. In addition, although the aging process in China is relatively slow, due to the large population base and the aging speed is gradually accelerating, the degree of aging varies in different regions.

According to a survey by the Health Information Resources Research Center of Huazhong University of Science and Technology (2), first of all, what the elderly need most is general health services and daily preventive health care; in terms of consumption expenditure, the food and medical expenses of the elderly occupy the top two daily consumption expenditures, followed by the provision of offspring and entertainment. Obviously, medical care Expenses have become a very important expense in the daily life of the elderly. Secondly, the results of the survey on the behavior of the elderly in seeking medical treatment show that 80% of the elderly will choose different medical institutions for medical treatment when they feel that they are mildly ill. The rest choose individual clinics and army/enterprise hospitals; and when they feel seriously ill, the vast majority of the elderly will go to medical institutions for medical treatment, and the most elderly people choose district, provincial and municipal hospitals for medical treatment. At the same time, in both cases, there is a situation of illness without medical treatment, and the economic situation is the biggest reason. When investigating the reasons why the elderly choose medical institutions, the following conclusions are drawn: the choice of medical institutions in the elderly's medical behavior is mainly affected by convenience, medical level, and medical expenses. The elderly have a clear preference for medical institutions with close distance/convenient transportation, high medical level, good reputation, good attitude of medical service personnel, reasonable service price, short waiting time, advanced equipment and complete facilities, and comfortable medical environment. The medical level is the most important factor affecting the elderly's choice of medical treatment, followed by reputation and service price. Other influencing factors can be summarized as institutional service level factors (3).

It is also a top priority to promote the development of community-based basic medical institutions. Due to the inconvenience of the elderly, the elderly should consider the convenience requirements more when choosing medical institutions for medical treatment. Therefore, the nearby community and street health service centers should be the places for the elderly to seek medical treatment. The first choice institution; however, the medical service level of primary medical institutions is relatively lower than that of large hospitals at the provincial and municipal levels, which cannot meet the needs of the elderly for the service level of medical institutions. There are various problems of “difficulty in seeing a doctor” such as long waiting times and long distances for medical treatment. In response to this problem, the health service level of primary medical institutions should be strengthened, and barrier-free passages should be set up for the elderly in each medical institution, so as to reasonably guide the flow of medical treatment choices of the elderly, and strive to alleviate the problem of “difficulty in seeing a doctor” in the elderly's medical treatment. On the other hand, the government should strengthen the management of medical services in various institutions, standardize the medical treatment process, control medical expenses, and use the basic drug system, etc., to form a reasonable medical price mechanism, and alleviate the problem of “expensive medical treatment” for the elderly (4).

### Community health service

Community health service is under the leadership of the government, community participation, and the guidance of higher-level health institutions, with people's health as the center, family as the unit, community as the scope, and demand as the orientation, with primary health institutions as the main body, and general practitioners as the main force (5) resources and professional technology, actively provide all residents of the community, especially women and children, disabled, elderly and other key service objects, establish health records, formulate community health plans, provide diagnosis and treatment services, prevent and treat chronic diseases, and conduct health education, etc. Basic health services are economical, convenient, comprehensive and effective. They aim to solve the main health problems in the community and meet the needs of basic health services. They are the foundation and core of the health system and an important part of community construction (6, 7).

With the increasing progress of science and technology and culture, in order to meet people's growing demand for basic health services, community health services are an effective means to adjust the rational distribution and allocation of health resources. During the transition period of the economic system, some new contradictions and new problems have emerged, which are mainly manifested in the following aspects: the rapid growth of drug costs and heavy personal burden; the large disparity in the allocation of health resources between urban and rural areas; the continuous decline of the efficiency of medical services; the aging population The process of transformation is accelerated. These problems also affect the health behavior of the elderly.

Community health service centers, as primary medical institutions, are an important part of urban health work and the basic link to achieve the goal of primary health care for everyone (8). It serves all residents in the community widely, and it is inclined to key groups such as the elderly, the weak, the sick and the disabled. For the elderly who are aged at home in the community, the convenience of medical treatment in the community plays an increasingly important role in the protection of their health and quality of life.

## Literature review

### Research on the demand for community health services at home and abroad

#### Current status of foreign research

Developed countries entered an aging society many years earlier than China. In the mid-1990s, the international community began to propose to minimize the number of elderly people changing old-age places, and to make the elderly live in the living environment they are familiar with as much as possible. To obtain long-term and continuous elderly care services, and advocate the establishment of a “continuous care” elderly care service system. Developed countries such as the United Kingdom, the United States, and Japan have successively established long-term care systems in line with their national conditions. The elderly health care service system is relatively complete, and the community elderly medical network framework with multiple levels of medical treatment, health care, and nursing has been relatively complete.

The United Kingdom has become a country with an aging population as early as 1929, and its pension model is mainly a community care model consisting of “In-community care” and “Community care” (9), the difference lies in whether the government directly intervenes. The former is a normative old-age care with government intervention, while the latter is a non-normative old-age care without direct government intervention, usually through blood or moral relationships, i.e. family members, neighbors, friends, charities and non-profit organizations. Organization, etc., to maintain (10).

The United States entered an aging society as early as around 1940, and now a relatively mature and comprehensive market-oriented elderly care service industry has been formed. It generally adopts the model of commercial insurance, emphasizing the right of the elderly to choose independently, and can freely insure according to their own needs. The main feature of the old-age care model in the United States is that under the market economy system, the government guides and supervises its policies, and develops long-term care into an industry. At present, a pension pattern with coexistence of various models such as PACE, HCBS, CMO, ASAP, and RCC (11) has been formed.

Japan's adoption of the Nursing Insurance Law first solved the worries for the elderly and their families economically, and the elderly care service industry has achieved great development. At the same time, it has also alleviated the “press bed” that hospitals deliberately choose to be hospitalized because the elderly want comprehensive care. In this way, the cost of medical and elderly care institutions is reduced, the medical expenses of the elderly are reduced, and the burden on the government and society is also reduced. The grassroots communities in Japan generally have small-scale, multi-functional and community-based service facilities, which are the hardware guarantee for home care. With the continuous development of Japan's long-term care insurance system, the academic community has made a systematic study of its development status, existing problems and reform directions. Anonymous believes funding sustainability can be addressed from a business perspective (12); Imai et al. believe that the advantage of the long-term care insurance system is that the elderly can obtain long-term professional medical care services in institutions other than hospitals, which satisfies the wishes of the elderly family for retirement (13). Shimizutani discussed the future reform trends of the long-term care insurance system, using data to study input costs, incentive mechanisms, etc. from a micro perspective (14).

#### Domestic research status

There are three basic pension models in China: family pension, institutional pension and community home-based pension (15). The increasingly severe aging trend has brought challenges to these three pension models. The traditional family structure has been transformed into a “4-2-1” structure, and the problem of insufficient motivation for the development of family pension is becoming more and more serious. Restricted by the lack of institutional resources and insufficient capital investment. Community-based home-based elderly care can provide the elderly with services such as life care, rehabilitation care and spiritual comfort. It can alleviate the pressure of children's care for the elderly and improve the quality of life of the elderly in their later years. However, (16) a major challenge faced by community-based home care is how to solve the increasingly poor health problems of the elderly at home.

In recent years, the local pilot work of the combination of medical care and nursing has provided empirical materials for the research. Beijing, Shanghai, Qingdao, Chongqing and other places have carried out practice and formed a model with local characteristics. Liu Shiyang conducted a survey on various medical and elderly care institutions in Beijing and put forward multi-level suggestions (17). Shen Wanwan explored the optimal mode of cooperation between Shanghai elderly care institutions and community health service centers (18). Starting from the background of Qingdao's medical and elderly care integration model, Li Jie analyzed the operation of Qingdao's medical and elderly care integration mode (19). Taking Banan District, Chongqing City as an example, Jing Sixia summed up the service model of combining medical care and elderly care in line with the actual characteristics of Southwest China (20). In 2017, General Secretary Xi Jinping also clearly pointed out in the report of the 19th National Congress of the Communist Party of China that it is necessary to implement a healthy China strategy, actively respond to the aging of the population, build a policy system and social environment for the elderly, filial piety and respect for the elderly, promote the combination of medical care and elderly care, and accelerate the cause of aging and industry development.

### A study on the medical treatment behavior of the elderly

#### Foreign research on the behavior of the elderly in seeking medical care

This paper divides the foreign research on medical treatment behavior into the following three aspects: theoretical research on medical treatment behavior, model research on medical treatment behavior, and research on influencing factors of medical treatment behavior.

Research on medical behavior theory research (21): The behavior of seeking medical treatment is a series of behaviors that people take the initiative to take medical measures when they feel unwell, in order to find the cause of the disease and reduce the harm caused by the pain. Research on the model of influencing factors of medical treatment behavior: foreign scholars mostly use discrete choice model to explore the choice behavior of resident medical institutions (22, 23) explored the relationship between Vietnamese residents' choice of different medical institutions and their participation in different medical insurance; Hanson et al. (24) discussed the influence of factors such as service quality, technical level, medical expenses and drug prices of medical institutions on patients' medical behavior; Wang et al. (25). A multivariate logistic model was used to explore the effects of insurance characteristics and residents' income on the patient's choice of medical treatment in three levels of medical institutions: village clinics, township health centers and county-level hospitals. Based on the above studies, it can be seen that most of the foreign studies on the impact of residents' medical treatment behavior use the regression model of related variables, especially the probit model and the logistic model are widely used (21). Foreign research on factors of medical seeking behavior can be roughly divided into the following three types: first, to explore the influence of medical institutions' technical level, service quality, medical price, medical distance and other factors on patients' medical seeking behavior choice; Secondly, the influence of family characteristics and personal characteristics of patients on the choice of medical institutions was discussed. Third, explore the influence of medical insurance participation on patients' choice of medical place.

#### Domestic research on medical treatment behavior of the elderly

In the domestic research on medical treatment behavior, in the early stage of research methods, mainly through data description and analysis, especially the influencing factors of patients' choice of medical institutions. Ren Xiaohui and others explored the influence of medical insurance participation on patients' choice behavior of medical institutions, initiative and timeliness of seeking medical treatment. Qian Dongfu took patients in economically backward remote rural areas as the research object, and applied a multivariate logistic model to analyze the influence of their choice of medical institutions and treatment methods on their medical behaviors (26). Zhang Bing and Wang Yiqiu conducted related research on the choice of residents' medical treatment behavior at the level of medical institutions, and found that when patients are seriously ill, they are more inclined to go to higher-level medical institutions for diagnosis and treatment. At this time, medical price factors have no significant impact on patients' choice of medical treatment behavior, and when the patient's physical fitness is good and suffers from minor or common diseases, the patient pays more attention to the medical price, and the price becomes a significant factor for the patient's choice of medical treatment behavior (27). Bao Yong et al. analyzed the medical treatment intention and influencing factors of residents in a community in Shanghai by compiling their own questionnaires (28). Jiang Jinqi applied a multi-factor logistic model to study the choice of medical treatment behavior of rural residents after illness, and found that in the early stage of the new rural cooperative medical system in 2004, the farmers who participated in the new rural cooperative medical insurance were more inclined to choose after illness. Go to a nearby clinic to seek medical treatment. However, by 2007, whether or not to participate in the new rural cooperative medical insurance has no significant impact on whether farmers go to the doctor when they become ill, but it increases the proportion of farmers who self-medicate (29).

Therefore, the research on the influencing factors of residents' medical treatment in China is mainly focused on the influencing factors of patients' choice of medical treatment, and patients' medical treatment behavior is mainly affected by material conditions.

### Research questions

This paper is based on the background of population aging to study the behavior of the elderly, especially the impact of the health behavior of the elderly on community health care. As we all know, the health problems of aging are complex issues involving multiple levels. The rapid development trend of “aging society” will inevitably lead to changes in the national disease burden, financing and investment patterns. At the same time, the current situation of health care, provision and utilization of health care services for the elderly in China is worrying. Compared with other countries, the health services for the elderly in China still need to be improved. What constructive measures can be taken? Finally, the social support system for elderly health services in China is lacking. China is in the process of building and improving the new medical reform medicine and public health system. Professional medical care resources for the elderly are even more scarce, and there is a lack of sociality outside other professional medical care institutions. Support institutions added that the sick elderly can only be cared for by their family members or hired social personnel, which directly increases the economic and psychological burden of the elderly family.

## Research design

### Research content

This article addresses the impact of older adults' behavior on community health care in the context of an aging world population. The general law of the behavior of the elderly is the main factor to promote the development of community medical care. According to different behaviors of the elderly, different measures should be taken to prescribe the right medicine, optimize the medical and health links, and comprehensively promote the medical alliance services. The hierarchical diagnosis and treatment model of referral, classification of acute and chronic diseases, and linkage between upper and lower levels” realizes family doctor contract signing and team building of general practitioners, provides convenient medical services for the elderly, improves the reform of the medical system, and implements “double sinking”, two “Upgrade” project to speed up the development of grass-roots community health service institutions (12). Through the analysis and discussion on the development strategy of community health service institutions in Hedong District, Tianjin, we can provide better medical security for home-based elderly care, promote the comprehensive strengthening of community health services, reduce the increasingly severe burden of aging, and achieve healthy elderly care.

### Research methods

**Figure 2:**
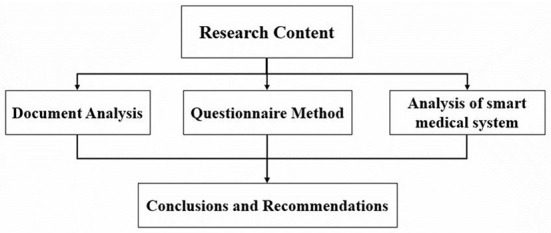
Research flow chart.

This paper analyzes the impact of elderly behavior on community health care under the background of aging (as shown in Figure 2) by comprehensively using literature analysis, questionnaire survey and computer intelligent medical system analysis, and provides suggestions for the realization of healthy old age and the development of community health care. Constructive advice.

#### Literature analysis method

Collect a large number of literature materials related to old-age care, medical care, community health services, and the combination of medical care and elderly care, organize and classify, read and research, understand existing domestic and foreign related theories and research results through literature materials, and sort out domestic The development of foreign community health service institutions provides a theoretical basis for the writing of the thesis.

#### Questionnaire survey method

A survey questionnaire was designed to understand the cognition, demand and use of community health service institutions among the elderly groups in six districts of Tianjin City as an example, and relevant conclusions were drawn through quantitative analysis of the questionnaire survey.

#### Computer intelligent medical system analysis method

Through the regional medical information platform for health records created in recent years, the most advanced Internet of Things technology is used to realize the interaction between patients and medical personnel, medical institutions, and medical equipment, and gradually achieve informationization. The smart system represented by smart access control and smart community conducts data analysis on the daily behavior patterns of the elderly on the big data platform, draws conclusions about the general behavior of the elderly, and gives corresponding measures.

### Innovations and shortcomings

Scholars at home and abroad have done a lot of research on the combination of medical and nursing care in recent years, and the theoretical results are relatively rich. After consulting a large number of literature and materials, they found that the focus of attention is mainly on how to break the system fortress and establish hospitals and sanatoriums that integrate medical and nursing care. In China, more attention is paid to the feasibility and policy implementation of the combination of medical care and elderly care. There are relatively few studies on how to develop community health service centers in order to provide better health services for the elderly at home in the community. This paper grasps the trend of the times, studies the age-appropriate development of community health service institutions as a breakthrough point, and proposes countermeasures from the perspectives of human resources, hardware facilities and system construction to promote the development of community health service centers.

Due to the limitation of human factors, this paper adopts the questionnaire survey method which is more convenient for sampling survey. The data results of the survey are based on the 365 questionnaires collected, which may lead to biased conclusions; and personal thinking is not comprehensive enough, which may also affect the quality of research. As a path study on the development of grass-roots community health service centers under the background of population aging, if time and samples are sufficient, I think this paper has more room for further discussion (30).

### Research questions and hypotheses

**P1: What are some constructive suggestions for improving the national health services for the elderly?**

This paper is based on the background of population aging to study the elderly behavior, especially the elderly health behavior on the community health care. As is known to all, the aging health problem is a complex problem involving multiple layers. The rapid development trend of “aging society” will inevitably lead to changes in the country's disease burden, financing and investment pattern. At the same time, the current situation of health care and medical insurance provision and utilization in China is worrying. Compared with other countries, China's health services for the elderly still need to be improved. What constructive measures can be taken? The lack of social support system of elderly health services in China, it is in the construction of the new health care medicine and public health system and perfect, professional medical care for elderly people more scarce resources, lack of social support besides other professional medical and health institutions complement, sick old man only by family members or hire social personnel's care, This directly aggravates the economic and psychological double burden of elderly families.

**H1:** The background of the aging population in the world and the lack of social support system for health services for the elderly in China.

**H2:** China's community healthcare system has increased physical security requirements based on an aging population.

**H3**: Smart health systems have a positive effect on the medical behavior of the elderly.

### Questionnaire method

#### Research object

The subjects of this study were the elderly in urban areas of Tianjin, and a sample survey was conducted on the living conditions, health conditions, and health service needs of the elderly over 60 years old in the community. A total of 400 questionnaires were sent out, and 365 were recovered, with a recovery rate of 91.25%, of which 365 were valid questionnaires.

A random sampling method was adopted to select six main urban areas in Tianjin, and three streets were randomly selected in each urban area for questionnaire surveys. The surveyors issued questionnaires to the respondents in a uniform manner with the same survey standards and in a one-to-one manner. At the same time questionnaire survey results are promised to the respondents confidentiality, will not disclose personal privacy.

#### Data processing

After collecting and arranging the data obtained from the questionnaire survey. Epidata was used to establish a database, double-checked and entered, and SPSS 19.0 software was used for statistical analysis of the data.

#### The basic situation of the data results

The respondents of this survey are the elderly over 60 years old, including 165 males and 200 females. The average survey age is 67.5 years old. The youngest is 61 years old and the oldest is 89 years old. 81% of the survey respondents are concentrated in the age group of 60. Between 72 years old. The educational level of the respondents is concentrated in primary school, junior high school, and high school, accounting for 28, 26, and 25% respectively. The illiterate and highly educated people are <21%. Most of the spouses of the respondents in this study are still alive, the proportion is higher than 75%. 48.77% of the elderly feel that their health status is average, as shown in Table 2 below, more than 37% of them feel that their health status is good, and only 11% of them feel that their health status is not good. Therefore, what the elderly need most is general hygiene services and routine preventive care. In terms of consumption expenditure, it can be seen from Table 3 below that food expenses and medical expenses occupy the top two positions in daily consumption expenditures, followed by the supply of offspring and entertainment. Obviously, medical expenses have become a very important part of the daily life of the elderly expenses.

**Table 2:** Individual self-perceived health status of the elderly.

**Perceived health status**	**Frequency (Number)**	**Percentage (%)**
Very good	45	12.33
Good	98	26.85
Generally	178	48.77
Not good	36	9.86
Very bad	5	1.37
Can't answer	3	0.82
Total	365	100

**Table 3:** Consumer spending of the elderly.

**Expenditures**	**Frequency (Number)**	**Percentage (%)**
Food expenses	361	98.90
Medical fees	312	85.48
Supply offspring	135	36.99
Entertainment	89	24.38
Housekeeping	69	18.90

A survey on the medical consumption behavior of the elderly in urban areas of Tianjin found that among 365 elderly people, 67.4% of the elderly chose to go to a medical institution for medical treatment when they were physically and mentally unwell, 30.7% of the elderly chose to go to the pharmacy to buy medicine first, and 1.9% of them chose to go to the pharmacy first of older adults choose not to treat at first. When choosing a medical institution for the first consultation, 33.2% of the elderly chose to go to a general clinic, 48.5% of the elderly chose to go to a community/township health center, 15.1% chose a county/district hospital, and 3.3% of the elderly chose to go to a hospital above the county level. When choosing a medical institution, the elderly mainly focus on two factors: convenience (48%) and reasonable cost (29%). Level (0.5%) and other aspects are considered less. In the choice of medicines, the elderly are more concerned about the efficacy (43.8%) and cost (36.2%) of medicines, while the word-of-mouth (9.9%), brand awareness (3.0%) and pharmacy or hospital recommendation (7.1%) of medicines are more concerned with the elderly. Less attention. The majority of seniors' healthcare decisions are made by themselves (55.1%), followed by partners (26.6%) or husband and wife (13.2%), and fewer seniors make decisions by children (5.2%).

It can be seen that, as a first-tier city in China, the consumption level of the elderly in Tianjin is obviously higher than that in other areas. However, there are still many elderly people who consider the cost factor when choosing medical institutions, and also consider the price when choosing medicines. Therefore, an obvious conclusion can be drawn. For the reform of social medical care, price fairness and even lowering the price of medical consumption are the top priorities, and making the elderly willing to spend money on medical care is the only way to reform. As show in Tables 4, 5.

**Table 4:** Descriptive table of medical consumption behavior of the elderly in urban areas of Tianjin (*n* = 365).

**Medical consumption behavior**		**Number**	**Composition ratio (%)**
The first choice for mental and physical discomfort	Go to a medical institution	246	67.4
	Go to the pharmacy	112	30.7
	Without treatment	7	1.9
First when sick medical institution	General clinic	121	33.2
	Community/township health center	177	48.5
	County/District Hospital	55	15.1
	Hospitals above the county level	12	3.3
Key Factors in Medical Institution Selection	Medical convenience	175	48.0
	Reasonable cost	106	29.0
	Good environment	17	4.7
	High medical skills	62	17.0
	Careful	3	0.8
	High level	2	0.6
Key Factors in Drug Selection	Curative effect	160	43.8
	Cost	132	36.2
	Word of mouth	36	9.9
	Brand awareness	11	3.0
	Pharmacy or hospital recommendation	26	7.1
Decision makers in healthcare	Own	201	55.1
	Companion	97	26.6
	Husband and wife	48	13.2
	Child	19	5.2

**Table 5:** The distribution of medical insurance participation of the elderly (*n* = 365).

**Type**	**Frequency (Number)**	**Percentage (%)**
Urban Employee Medical Insurance	121	33.15
Basic Medical Insurance for Urban Residents	132	36.16
New rural cooperative medical care	68	18.63
Commercial medical insurance	25	6.85
None	19	5.21

The questionnaire survey involves the participation of the elderly in medical security. From the survey results, the vast majority of the elderly enjoy medical security, and only a very small number of the elderly do not have medical security. Among the 365 valid survey respondents, specifically: 121 people participated in the medical insurance for urban employees, accounting for 34.38 of the total number; 132 people participated in the basic medical insurance for urban residents, accounting for 37.50% of the total number; There are 68 people with cooperative medical care, accounting for 19.32% of the total number; 25 people participating in commercial medical insurance, accounting for 5.68% of the total number; 19 people without any insurance, accounting for 3.12% of the total number. Among them, the proportion of medical insurance for urban employees and medical insurance for urban residents is similar, and the proportion is relatively high, the proportion of participating in commercial insurance is low, and the proportion without medical insurance is the least.

When choosing a medical institution for the first consultation, 77% of the elderly chose to go to community township health centers and general clinics, and only 23% of the elderly did not receive community health services, as shown in Table 6. This shows that when encountering health problems, community health care is the first choice for the vast majority of the elderly, and the quality of community health care directly determines the health behavior of the elderly.

**Table 6:** Frequency distribution of elderly people receiving community health services (*n* = 365).

**Whether receiving community health services**	**Frequency (Number)**	**Percentage (%)**
Yes	281	76.99
No	84	23.01

### Smart medical system analysis

With the rapid development of technologies such as big data, Internet+, and 5G, concepts such as smart medical care, “big health”, and medical big data have appeared frequently, and have attracted great attention in the field of smart medical care at home and abroad (31). The core of smart health management, one of the important components of smart medical care, lies in the information data and electronic health records of patients (32). However, the inadequacy of current health management is limited by traditional medical methods, which can only go to a doctor or participate in a physical examination at a fixed time. Therefore, the important physiological parameters of patients cannot be monitored in real time and continuously, which leads to delays in discovering potential diseases and affects the most the best time for treatment. In addition, there are still many people in China who are not aware of basic chronic diseases such as cardiovascular and other basic chronic diseases, and the treatment rate is even lower. Almost more than half of the population has not implemented effective prevention and treatment (33). Basic chronic diseases are usually closely related to physiological parameters such as blood pressure, blood oxygen, body temperature, and respiration. In order to prevent and treat chronic diseases, it is very meaningful to continuously measure such physiological parameters. At the same time, the monitoring of human body posture can also be particularly important for the health management of the elderly (34).

In recent years, medical technology based on artificial intelligence has achieved rich research results, and has also occupied a very important position and role in the development of smart medical care, and is further opening a new era of smart medical care. Compared with the shortcomings of traditional medical management methods, which are backward and the measurement accuracy of physiological parameters is not high, the smart medical analysis method mentioned in this article is to design a system with embedded AI technology. access control technology. Through the core hardware platform, the received signals are subjected to quality evaluation, filtering, denoising, enhancement, feature extraction, heartbeat classification and other work processing, and then uploaded to the cloud through the module for health management of elderly users. At the same time, the system can also complete the interaction between users and doctors, and can further establish an individualized physiological parameter database, so as to realize the dynamic tracking management and disease prevention of diseases of the elderly.

#### Design of system acquisition module

**Figure 3:**
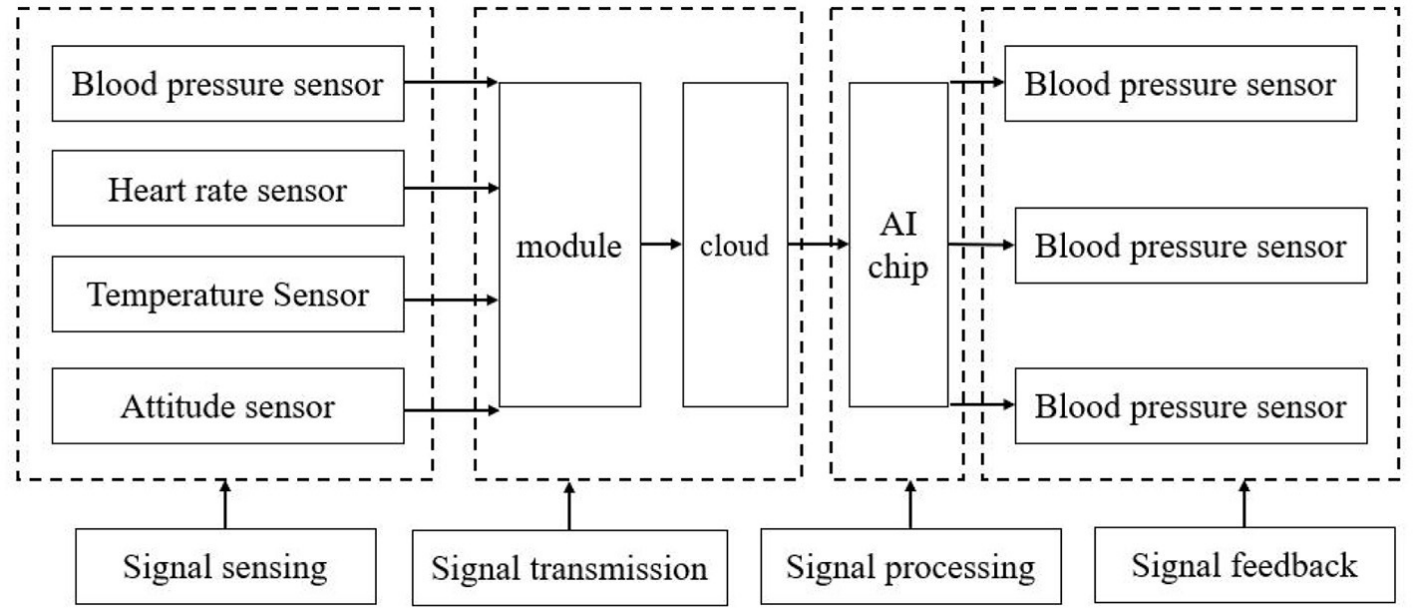
System overall frame diagram.

The system acquisition module designed in this paper is shown in Figure 3. It mainly includes four parts: information perception, information transmission, signal processing, and signal feedback. The sensing part mainly includes sensors to collect the physiological parameters of the elderly; signal transmission mainly uploads the collected and preprocessed data to the terminal or cloud server through the module; Next, AI algorithms are required to evaluate the quality of the received signals, Filtering, denoising, enhancement, feature extraction, cardiac beat classification and other work processing; finally, the information processed by AI algorithm is transmitted to users and doctors, ECG database and health management center, and can be continuously tracked.

#### User usage mode

The user usage mode is a mode in which doctors use the system to diagnose patients, and patients can log in to the system to view their own diagnosis results. In order to realize this mode, the doctor needs to first receive the patient through the registered diagnosis module. If the selected patient has not applied for the collection terminal, he needs to fill in the patient's disease information, make a diagnosis, and provide the corresponding treatment plan. If the disease is serious, the doctor needs to guide the patient to register with the system and apply for the collection terminal; if the disease is mild, the diagnosis can be ended and the next patient can be selected until the end of working hours. If a patient needs to apply for a collection terminal, they need to enter the patient ID number in the information module to view their historical diagnosis data, and then use the medical auxiliary module to diagnose and obtain the treatment plan, enter it into the diagnosis dialog box and upload it to the database. In this way, patients can log in to the system to view the diagnosis results independently (35).

The overall frame diagram of the system reflects the data interaction mode of the smart medical system. The elderly can automatically collect the required data (heart rate, blood pressure, body temperature, etc.). Data collection system, ideally, the cloud will automatically analyze the health status of the elderly based on the collected data on the day. If any problem is found, it will alert the elderly to pay attention and upload the problem data to the community medical center for doctors to analyze and provide medical measures. In this way, the elderly can know their health status without leaving home. If necessary treatment is needed, they will also be recommended to the nearest community medical and health service center for diagnosis and treatment, so as to truly achieve old age with medical care.

## Results and discussion

From the perspective of literature analysis, from the perspective of questionnaire survey, and from the perspective of smart medical care, this article studies the impact of the behavior of the elderly on community health care in many aspects. The following conclusions can be drawn:

(1) The vast majority of the elderly receive treatment at community health service centers, accounting for 77% of the surveyed.
(2) The two factors of convenience for medical treatment and reasonable cost are the main considerations for the elderly to choose a medical place. The elderly prefer to seek medical treatment nearby, and they also try to be frugal in consumption, and do not like to go to expensive medical places for consumption.
(3) The elderly who are close to the average think their physical health is average, and many elderly people think that their body is in a sub-healthy state.
(4) The smart medical system can bring great convenience to the elderly and community health care, and can connect the two more closely.

Based on the above conclusions, combined with the impact of the elderly's behavior on community health care, the following suggestions are given:

(1) Improve the preferential medical treatment system for the elderly, so that the elderly can enjoy better medical conditions with less money;
(2) Optimize and adjust the industrial structure of the geriatric medical industry; hospitals change their management mode, implement refined management, and optimize and adjust the medical service structure focusing on medical services for the elderly.
(3) It is necessary to strengthen the supervision and management of the geriatric medical market and improve the consumption environment of the geriatric medical market.
(4) Promote the smart medical system, so that more elderly people can enjoy the dividends of science and technology, so that the elderly and social medical care are more suitable.

## Research conclusion and discussion

This article analyzes the characteristics of the world's aging population, starting with the trend of the world's aging population and the era of China's aging population. The article also researches the health care needs and behaviors of the elderly, and it can be seen that community health care is the key factor, and the availability of a good community health care system is directly related to the choice of health care behaviors of the elderly. The author also introduces the concept of community health service organization, which defines community health care more accurately.

In the content of the literature research section, the author conducts research from two perspectives, firstly, research on the demand side of community health services at home and abroad, in fact, research on the health care seeking behavior of the elderly at home and abroad, dissecting the material and intrinsic reasons.

The main core part of the article is the questionnaire research section, based on the results of the research and combined with the research questions and hypotheses we have some conclusions as follows.

(1) The majority of the elderly people accept to go to the community health center for treatment, accounting for 77% of the surveyed people.
(2) Convenience and reasonable cost are the two main factors that elderly people consider when choosing a place to go for medical treatment, and they prefer to seek medical treatment nearby and spend money as frugally as possible, and do not like to spend money in expensive medical places.
(3) Nearly the average elderly people think their health condition is average, and many of them think their body is in a subhealthy state.
(4) Smart medical system can bring great convenience to the elderly and community health care, and can link the two more closely together.

Regarding Q1, based on the above findings, the following recommendations are given in the context of the impact of older adult behavior on community health care.

(1) Improving the system of medical benefits for the elderly so that they can enjoy better medical conditions with less money.
(2) Optimize and adjust the industrial structure of the geriatric medical industry; hospitals change their management mode, implement fine management, and optimize and adjust the structure of medical services with a focus on medical services for the elderly.
(3) Strengthen the supervision and management of the geriatric medical market and improve the consumption environment of the geriatric medical market.
(4) Promote the smart medical system, so that more elderly people can enjoy the dividends of technology, and make the elderly more relevant to social healthcare (36–38).

## Outlook

This article has been studied from several aspects. The literature survey method gives an understanding of the research on the behavior of the elderly at home and abroad in recent years as well as the research on the function of community health service centers; the questionnaire survey method takes a certain region as an example and speaks with data very strongly to convince the readers, but there are still certain shortcomings (39, 40): firstly, the questionnaire survey method can have more sample space in order to come up with more general laws; secondly, intelligent medical care is a good method, but the article still lacks some key technical means, which can be combined and analyzed by professional software afterwards to make the article structure more complete.

## Data availability statement

The original contributions presented in the study are included in the article/supplementary material, further inquiries can be directed to the corresponding author.

## Author contributions

Conceptualization, software, and writing-original draft preparation: JZ and LW. Methodology: JZ and KG. Data curation: KG. Writing—review and editing: LW, JZ, and KG. Supervision: JZ. All authors have read and agreed to the published version of the manuscript.

## Funding

This work was supported by Wenzhou City Characteristic Advantage Professional Group Construction Project.

## Conflict of interest

The authors declare that the research was conducted in the absence of any commercial or financial relationships that could be construed as a potential conflict of interest.

## Publisher's note

All claims expressed in this article are solely those of the authors and do not necessarily represent those of their affiliated organizations, or those of the publisher, the editors and the reviewers. Any product that may be evaluated in this article, or claim that may be made by its manufacturer, is not guaranteed or endorsed by the publisher.
